# Molecular Cloning and Yeast Expression of Cinnamate 4-Hydroxylase from *Ornithogalum saundersiae* Baker

**DOI:** 10.3390/molecules19021608

**Published:** 2014-01-28

**Authors:** Jian-Qiang Kong, Di Lu, Zhi-Biao Wang

**Affiliations:** State Key Laboratory of Bioactive Substance and Function of Natural Medicines & Ministry of Health Key Laboratory of Biosynthesis of Natural Products, Institute of Materia Medica, Chinese Academy of Medical Sciences & Peking Union Medical College, Beijing 100050, China

**Keywords:** cinnamic acid 4-hydroxylase, *Ornithogalum saundersiae*, phenylpropanoid, OSW-1

## Abstract

OSW-1, isolated from the bulbs of *Ornithogalum saundersiae* Baker, is a steroidal saponin endowed with considerable antitumor properties. Biosynthesis of the 4-methoxybenzoyl group on the disaccharide moiety of OSW-1 is known to take place biochemically via the phenylpropanoid biosynthetic pathway, but molecular biological characterization of the related genes has been insufficient. Cinnamic acid 4-hydroxylase (C4H, EC 1.14.13.11), catalyzing the hydroxylation of *trans*-cinnamic acid to *p*-coumaric acid, plays a key role in the ability of phenylpropanoid metabolism to channel carbon to produce the 4-methoxybenzoyl group on the disaccharide moiety of OSW-1. Molecular isolation and functional characterization of the *C4H* genes, therefore, is an important step for pathway characterization of 4-methoxybenzoyl group biosynthesis. In this study, a gene coding for *C4H*, designated as *OsaC4H*, was isolated according to the transcriptome sequencing results of *Ornithogalum saundersiae*. The full-length *OsaC4H* cDNA is 1,608-bp long, with a 1,518-bp open reading frame encoding a protein of 505 amino acids, a 55-bp 5′ non-coding region and a 35-bp 3'-untranslated region. *OsaC4H* was functionally characterized by expression in *Saccharomyces cerevisiae* and shown to catalyze the oxidation of *trans*-cinnamic acid to *p*-coumaric acid, which was identified by high performance liquid chromatography with diode array detection (HPLC-DAD), HPLC-MS and nuclear magnetic resonance (NMR) analysis. The identification of the *OsaC4H* gene was expected to open the way to clarification of the biosynthetic pathway of OSW-1.

## 1. Introduction

**Figure 1:**
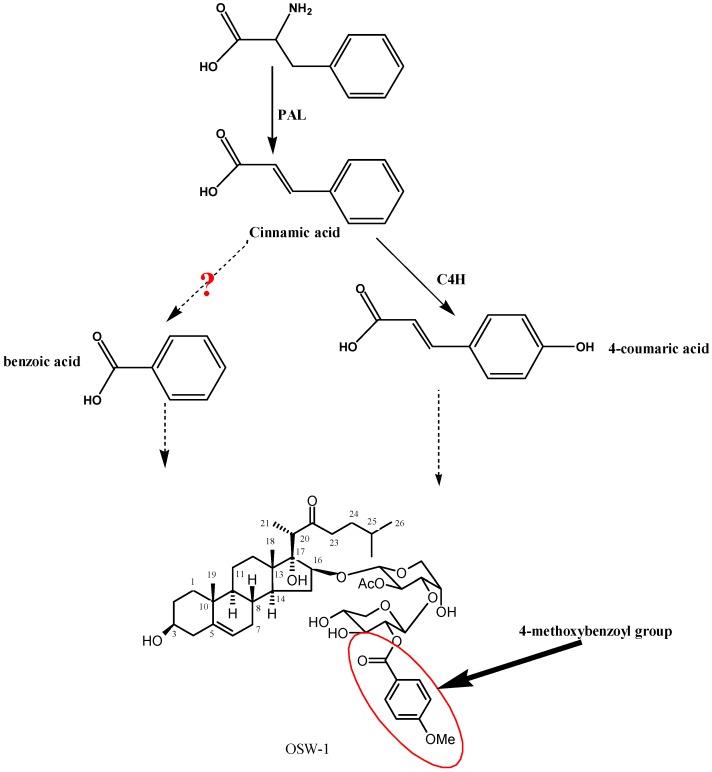
Putative pathway of OSW-1 biosynthesis.

OSW-1 (3β,16β,17α-trihydroxycholest-5-en-22-one 16-*O*-{*O*-[2-O-(4-methoxybenzoyl)-β-d-xylo- pyranosyl]-(1→3)-2-*O*-acetyl-α-l-arabinopyranoside}, Figure 1), was isolated from the bulbs of *Ornithogalum saundersiae* Baker (*O. saundersiae*) a perennial plant of the lily family cultivated in Southern Africa [1,2]. OSW-1 is about 10-100 times more cytotoxic than clinically applied anticancer agents such as mitomycin C, adriamycin, cisplatin, camptothecin, and paclitaxel, but has a low toxicity towards normal cells [1]. This potency, in combination with a unique mechanism of action and selectivity toward malignant tumor cells, gives OSW-1 and its analogues great potential as anticancer agents. Owing to the low content in the plant and laborious synthesis, the druggability study of OSW-1 had made slow progress since its first discovery in 1992 [2]. It is necessary to search for an alternative method of large scale production of OSW-1. Therefore, a thorough understanding of the biosynthetic pathway and characterization of the involved enzymes are important for the biological production of OSW-1 in a more economical way, such as using metabolic pathway engineering and synthetic biology. OSW-1 is characterized by the attachment of a disaccharide containing a *p*-methoxybenzoyl group to the C-16 position of the steroid aglycone. According to the previous SAR studies, the disaccharide moiety is important for the cytotoxicity, as removal of the acetyl (Ac) and the 4-methoxybenzoyl (MBz) groups on the disaccharide moiety decreased the activity about 1,000-fold [1,3]. Biosynthesis of the 4-methoxybenzoyl group on the disaccharide moiety of OSW-1 is known to take place biochemically by the phenylpropanoid biosynthetic pathway (Figure 1), but molecular biological characterization of related genes has not been fully achieved.

Cinnamic acid 4-hydroxylase (also known as cinnamate 4-hydroxylase, C4H, EC 1.14.13.11) is the second gene of phenylpropanoid pathway and a member of cytochrome P450 family (CYP73A) [4,5,6,7,8]. C4H catalyzes the hydroxylation of *trans*-cinnamic acid to *p*-coumaric acid, a precursor of 4-methoxybenzoyl (MBz) groups on the disaccharide moiety of OSW-1 [8,9,10]. Molecular isolation and functional characterization of *C4H* genes, therefore, is an important step in the characterization of the biosynthetic pathway of OSW-1 [11,12,13,14]. Previously, many putative *C4H* genes had been isolated from higher plants, such as Korean black raspberry [15], *Brassica napus* [16], *Catharanthus roseus* [17], poplar (*Populus trichocarpa* x *Populus deltoides*) [18] and *Helianthus tuberosus* [19,20,21]. However, only limited information is available on C4H and other enzymes of the phenylpropanoid biosynthesis in *Ornithogalum saundersiae*.

In the current study, a unigene annotated to *C4H* homology was acquired according to the transcriptome sequencing of *O. saundersiae*. A full-length cDNA, designated *OsaC4H*, was isolated from *O. saundersiae*. A comprehensive and precise functional analysis was then carried out. This is the first report of gene isolation and functional characterization of *OsaC4H* from *O. saundersiae*.

## 2. Results and Discussion

### 2.1. Transcriptome Analysis of OsaC4H Homology

OSW-1 is a cholestane saponin, featuring the attachment of a disaccharide to the C-16 position of the steroid aglycone. Biogenetic analysis showed there were at least five kinds of enzymes responsible for OSW-1 biosynthesis, that is steroid pathway enzymes resulting in OSW-1 aglycone formation, P450 hydroxylase able to add hydroxyl groups to the positions 3, 16 and 17 of the OSW-1 aglycone, a glycosyltransferase involved in disaccharide moiety attachment to the 16-OH of the OSW-1 aglycone, acyltransferases catalyzing introduction of the acetyl and the 4-methoxybenzoyl groups on the disaccharide moiety, and phenylpropanoid biosynthetic pathway enzymes converting aromatic amino acids to a 4-methoxybenzoyl group (Figure 1). A total of more than 40 enzymes were deduced to be involved in OSW-1 biosynthesis. It will take much more time to isolate and further functionally characterize all of these genes by conventional molecular biology technologies. Thus, it is particularly important to apply a high-throughput method, allowing for drastically quicker and cheaper gene discovery, leading towards a far more comprehensive view of the biosynthetic pathway of OSW-1. The advent of next-generation sequencing approaches such as transcriptomic analysis provides a platform, which has been proved to be critical in speeding up of the identification of a large number of related genes of secondary metabolite products. In the present investigation, 210,733 contigs and 104,180 unigenes were acquired from transcriptome *de novo* assembly. These congtigs and unigenes sequences were firstly aligned by Blast X to protein databases like nr, Swiss-Prot, KEGG and COG (e-value < 0.00001). KEGG annotation analysis indicated there were 1,958 unigenes involved in biosynthesis of secondary metabolites. Further batch alignment results revealed about 40 contigs and unigenes were annotated to be responsible for the phenylpropanoid biosynthetic pathway. Of them, one unigene, namely unigene 26946, showing high similarity with C4Hs was retrieved. Unigene 26946 was 1,608 bp long and was selected for further bioinformatics analysis and functional characterization.

**Figure 2:**
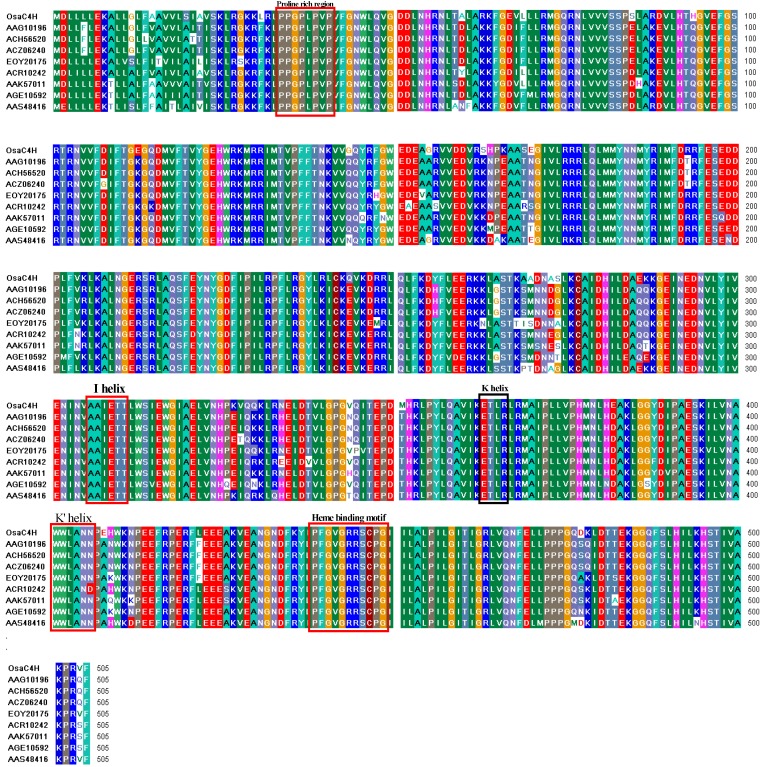
Alignment of the deduced full-length amino acid sequence of OsaC4H with other known C4H reported in reported in *Allium cepa* (AAS48416), *Gossypium arboreum* (AAG10196), *Theobroma cacao* (EOY20175), *Canarium album* (ACR10242), *Gossypium hirsutum* (ACZ06240, ACH56520), *Citrus x paradise* (AAK57011) and *Lonicera japonica* (AGE10592). P450 specific consensus sequences are boxed.

### 2.2. Bioinfomational Analysis of Unigene 26946

The NCBI on-line Blast X analysis [22,23,24] showed that unigene 26946 contained a sequence encoding a putative C4H. As predicted by the open reading frame (ORF) finder, the maximum ORF of the unigene 26946 is 1,518 bp long, encoding a 505 amino acid polypeptide. The theoretical molecular mass and pI of putative polypeptide are 57.8 kDa and 9.22, respectively. The instability index (II) is computed to be 45.70, which classifies the protein as unstable in a test tube. BLAST P in NCBI and multi-alignment analysis by Clust X indicated the deduced polypeptide was with highly identity to C4H reported in *Allium cepa* (AAS48416), *Gossypium arboreum* (AAG10196), *Theobroma cacao* (EOY20175), *Canarium album* (ACR10242), *Gossypium hirsutum* (ACZ06240, ACH56520), *Citrus x paradise* (AAK57011) and *Lonicera japonica* (AGE10592) sharing a similarity of 88%–89%, respectively (Figure 2).

There are three transmembrane domains in the deduced polypepetide, which indicates the polypeptide is membrane binding protein. Signal peptide prediction result indicated there was no signal peptide in the predicted peptide, which shows the protein is not an excreting protein, a conclusion in accordance with the transmembrane domain prediction result. 

Secondary structure and domain prediction revealed that deduced sequence contained 56.44%, 34.85% and 8.71% coil, α-helix, and β extended structures, respectively. Further domain prediction showed the target sequence harbored a P450 specific domain (between 32–503 aa). A comparative modeling of 3D model of OsaC4H was performed at ExPASy using SWISS-MODEL and the template for modeling was 3D structure of human P450 (PDB NO:3tbg). The model covered the 32–503 amino acids of the putative polypeptide (data not shown).

### 2.3. Cloning and Analysis of Full-Length Gene Encoding OsaC4H

**Figure 3:**
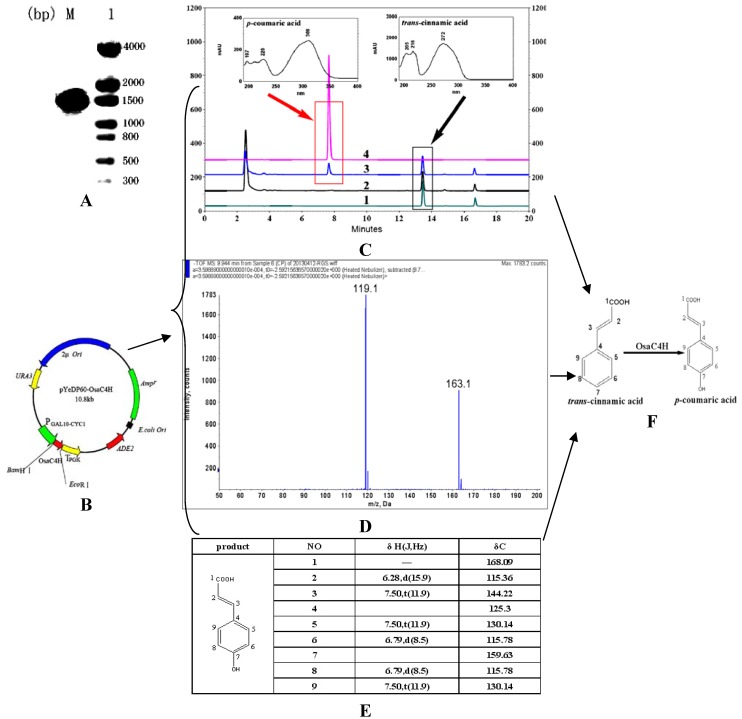
(**A**) Nested PCR analysis of *OsaC4H* gene (1). M represents DNA molecular marker DL2000. (**B**) The plasmid map of pYeDP60-OsaC4H. (**C**) HPLC analysis of reaction products from WHT[pYeDP60OsaC4H] (3) and WHT[pYeDP60] (2) using *trans*-cinnamic acid as the substrate. 1 and 4 refer to HPLC analysis results of standard *trans*-cinnamic acid and *p*-coumaric acid, respectively. Detection was set at 309 nm. (**D**) ESI-MS of *p*-coumaric acid in negative mode with singly charged pseudomolecular ion [M−H] at *m/z* 163.1. (**E**) Two-dimensional 600 MHz spectra of the product from *trans*-cinnamic acid catalyzed by OsaC4H. (**F**). Hydroxylation of *trans*-cinnamic acid to *p*-coumaric acid catalyzed by OsaC4H.

A full-length cDNA with 1,518 bp long was isolated from *O. saundersiae* by nested PCR without application of traditional molecule technologies like PCR amplification of conserved sequences by degenerate primers and RACE (Figure 3A). Sequencing verified that the cDNA sequence was identical with the result from transcriptome sequencing, which means a *bona fide C4H* gene *in planta*. All diagnostic features of the primary structure for cytochrome P450 enzymes, including subfamily CYP73A, are present in the putative sequence. Following the N-terminal anchoring sequence, a proline- rich region (consensus sequence (P/I)PGPx(G/P)xP) is present, which is supposed to destabilize the α-helix and produce kinks to optimally orient the enzyme with regard to the membrane. A conserved heme binding motif PFGVGRRSCPG (the consensus sequence in P450s is PFGXGRRXCXG) was also found near the C-terminus. In addition, some conserved helices like I helix (AAIETT), K helix (ETLR) and K’ helix (AWWLANN) were identified in the target P450 sequence. Furthermore, the deduced hydroxylase also possesses the residues N302 and I371 supposed to form hydrogen bonds and interact hydrophobically with the anionic site or the aromatic ring of cinnamic acid, and K484 that is required to spatially orient the substrate during or after the reaction (Figure 2). Therefore, the gene cloned in this study was designated as *OsaC4H* and the sequence information was deposited in the GenBank database (accession number KF741224). Then the *OsaC4H* gene was cloned by the In-Fusion method into *S. cere* visiae vector pYeDP60 resulting in heterologous plasmid pYeDP60- OsaC4H (Figure 3B).

### 2.4. Functional Characterization of Recombinant OsaC4H

Tremendous sequences were yielded by transcriptome sequencing. Thus, how to fast one can identify the biological function of these genes is an essential step in the characterization of the OSW-1 biosynthesis pathway. As an important enzyme in the formation of the 4-methoxybenzoyl group on the disaccharide moiety of OSW-1, the quick functional identification of OsaC4H exemplifies the characterization of other genes. Hence, a more simple high-pressure method was applied in the present study, which disrupts the yeast cells without tedious microsome preparation. Then varied substrates, such as *trans*-cinnamic acid, *p*-coumaric acid, caffeic acid, ferulic acid and sinapic acid, were added into the crude enzyme system to assess the enzymatic activity of OsaC4H. HPLC-DAD results showed that when the substrate *trans*-cinnamic acid was incubated with the engineered yeast WHT [pYeDP60OsaC4H] containing pYeDP60-OsaC4H, a new product peak was generated (Figure 3C). UV-Vis spectra of the new product were identical with those of a standard compound reported early [25]. No products, however, were found in the reaction systems with *p*-coumaric acid, caffeic acid, ferulic acid and sinapic acid as substrates (data not shown). LC-MS analyses of *trans*-cinnamic acid and the corresponding product displayed their [M−H]^−^ ions at *m/z* 147 and 163, corresponding to the calculated mass for *trans*-cinnamic acid and the hydroxylated cinnamic acid (Figure 3D). To further study the structure of the hydroxylated cinnamic acid, 8 mg of purified product was isolated by HPLC and subjected to NMR. The full assignment of the product is listed in Figure 3E. All the above evidence indicated that the hydroxylated cinnamic acid is *p*-coumaric acid, which meant the OsaC4H is a *bona fide* cinnamate 4-hydroxylase converting cinnamic acid into *p*-coumaric acid (Figure 3F).

### 2.5. Molecular Evolution Analysis of OsaC4H

**Figure 4:**
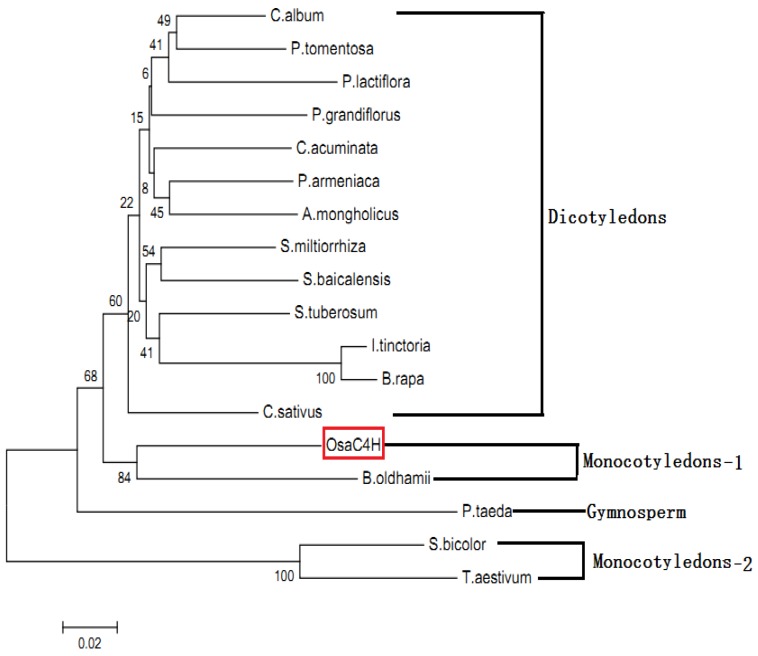
Phylogenetic tree illustrating the evolutionary relationships between OsaC4H and other C4Hs reported in *C. album* (*Canarium album*, ACR10242), *P. lactiflora* (*Paeonia lactiflora*, AGG55322), *S. miltiorrhiza* (*Salvia miltiorrhiza*, ABC75596), *S. tuberosum* (*Solanum tuberosum*, ABC69046), *C. acuminate* (*Camptotheca acuminate*, AAT39513), *I. tinctoria* (*Isatis tinctoria*, ADG43134), *B. rapa* (*Brassica rapa*, BAI77480), *S. baicalensis* (*Scutellaria baicalensis*, ADN32769), *P. armeniaca* (*Prunus armeniaca*, AEA02458), *A. mongholicus* (*Astragalus mongholicus*, AEH68208), *P. grandiflorus* (*Platycodon grandiflorus*, AEM63672), *C. sativus* (*Cucumis sativus*, CAK95273), *P. tomentosa* (*Populus tomentosa*, AFZ78542), *B. oldhamii* (*Bambusa oldhamii*, ACZ73612), *S. bicolor* (*Sorghum bicolor*, AAK54447), *T. aestivum* (*Triticum aestivum*, AAG17469) and *P. taeda* (*Pinus taeda*, AAD23378). The neighbor-joining tree was constructed using amino acid sequences through MEGA 5.05 with 1,000 boots trapped value support and a Poisson correction. The bootstrap values are indicated at the branch points. The scale bar represents 0.02 amino acid substitutions per site.

To investigate the evolutionary relationships among OsaC4H and C4Hs from other plant species, the phylogenetic tree was constructed using a neighbor-joining method. As reported in Figure 4, the phylogenetic tree was grouped into four main branches, including dicotyledons, monocotyledons-1, monocotyledons-2 and gymnosperm species. The result showed that OsaC4H was clustered into monocotyledons-1 species C4Hs, with a closest relationship with C4H from *B. oldhamii* (*Bambusa oldhamii*, ACZ73612).

## 3. Experimental

### 3.1. Substrates, Chemicals and Enzymes

*trans*-Cinnamic acid, *p*-coumaric acid, caffeic acid, ferulic acid and sinapic acid were obtained from Sigma-Aldrich Co. LLC (St. Louis, MO, USA). In-Fusion^®^ HD Cloning Kit and Restriction enzymes were purchased from Takara Shuzo Co. Ltd. (Kyoto, Japan). KOD Plus Taq DNA polymerase was purchased from Toyobo Co. Ltd (Osaka, Japan). All other fine chemicals are analytical grade. 

### 3.2. Strains and Plasmids

pEASY^TM^-T1 vector was from TransGen Co. Ltd (Beijing, China). The *E. coli* strain Trans1-T1 (TransGen) was used as a bacterial host for recombinant plasmid amplification. The strain was grown in Luria-Bertani medium (10 g·L^−1^ bacto-tryptone, 5 g·L^−1^ bacto-yeast extract, 10 g·L^−1^ NaCl) supplemented with ampicillin (100 μg·mL^−1^) when required for selection.

The yeast expression vector pYeDP60 and *Saccharomyces cerevisiae* strain WHT (MATa; ade2-1; his3-11,-15; leu2-3, -112; ura3-1; trp1-1) were kindly provided by Prof. Werck-Reichhart (Institute of Plant Molecular Biology, Strasbourg, France). pYeDP60 is a 2 µm plasmid with *GAL10-CYC1* promoter, *URA3* and *ADE2* marker. WHT is an engineered yeast in which the endogenous P450 reductase was replaced with the coding sequence of a P450 reductase from Jerusalem artichoke (*Helianthus tuberosus*), HTR1, under the control of the galactose-inducible promoter *GAL10-CYC1*. All yeast strains were grown either in the non-selective YPD medium (10 g·L^−1^ yeast extract, 20 g·L^−1^ bactopeptone, 20 g·L^−1^ glucose) or in the selective SD medium (0.7% yeast nitrogen base without amino acids, 0.1% casamino acids, and 2% glucose) with adenine and uracil dropped out where appropriate at 30 °C.

### 3.3. Plant Materials

The bulbs of *O. saundersiae* were grown under sterile conditions on 6,7-V medium [26] in culture room with temperature of 22 ± 2 °C and 16 h photoperiod. Prior to RNA isolation, experimental bulbs were collected and ground with a mortar and pestle under liquid nitrogen.

### 3.4. Transcriptome Sequencing and Analysis

RNA extraction was performed according to the standard protocol of RNeasy Plant Mini Kit (Qiagen, Valencia, CA, USA) with glass bead disruption. Total RNA was first assessed for quality on a Bioanalyzer 2100 using Nano 6000 LabChip (Agilent Inc., Santa Clara, CA). A complementary DNA (cDNA) sequencing library was prepared from the total RNA using an mRNA-seq Sample Preparation Kit (Illumina, San Diego, CA, USA) following the manufacturer’s protocol. Briefly, poly(A) mRNA was first purified using beads with oligo(dT). Then, the mRNA was fragmented into small pieces using fragmentation buffer. Taking these short fragments as templates, random hexamer-primer and reverse transcriptase (Invitrogen, Carlsbad, CA, USA) were used to synthesize first-strand cDNA. And then second-strand cDNA was synthesized. Short fragments were purified with QiaQuick PCR extraction kit and resolved with EB buffer for end reparation and adding poly (A). After that, the short fragments were connected with sequencing adapters. And, after the agarose gel electrophoresis, the suitable fragments were recovered as templates for the following PCR amplification. At last, the resultant cDNA library could be sequenced using Illumina HiSeq™ 2000 (Illumina). Short nucleotide reads obtained via Illumina sequencing were assembled by the Trinity software to produce error-free, unique contiguous sequences (contigs). Then, these contigs were connected to acquire non-redundant unigenes, which could not be extended on either end. Unigene sequences were aligned by Blast X to protein databases like nr, Swiss-Prot, KEGG and COG (e-value < 0.00001), and aligned by Blastn to nucleotide databases nt (e-value < 0.00001), retrieving proteins with the highest sequence similarity with the given unigenes along with their protein functional annotations. A candidate cinnamate 4-hydroxylase unigene, unigene26946, was identified by sequence homology to known cinnamate 4-hydroxylase.

### 3.5. Bioinformatics Analyses

The obtained cinnamate 4-hydroxylase candidate unigene 26946 was analyzed using online bioinformatics tools from NCBI and ExPASy. Blast X was done at the NCBI server using the unigene 26946 as the searcher. ORF finding was performed by the on-line program [27]. The amino acid sequence of the resultant ORF was deduced and analyzed with ProtParam tool [28]. The protein family of the deduced amino acid sequence was further predicted by Pfam [29,30]. The transmember domains were predicated using TMpred software [31]. Analysis of signal peptide was done by Signal P4.1 tool [32]. The on-line tool TargetP 1.1 was used for cell location [33]. A comparative modeling of 3D model of OsaC4H was performed at ExPASy using SWISS-MODEL.

### 3.6. Generation of Full-length OSaC4H cDNA

Since the assembled sequences were products of *de novo* assemblies, they were considered prone to error. To confirm that the sequence represented true gene product, experimental verification was performed by designing gene-specific primers (Table 1) for the OsaC4H full length sequence and verifying the identity of amplified product by sequencing of the PCR amplimers.

**Table 1:** Primers used in gene isolation and plasmid construction.

Primers	Sequences(5'-3')
Fcin450-1	5'- tcttcttcgc ccaagatatc aat -3'
Rcin450-1	5'- caagcggagc aatcaaaggg aaac -3'
Fcin450-2	5'- atggacctcc tcctcctaga g -3'
Rcin450-2	5'- ttagaacacc ctaggtttgg c -3'
FYeDP60cin450	5'- ctaaattacc ggatccatgg acctcctcct cctagag -3'
RYeDP60cin450	5'- gatcccccgc gaattcttag aacaccctag gtttggc -3'

A standard nested PCR approach consisting of the Fcin450-1/Rcin450-1 primer set (external primers, Table 1) and the Fcin450-2/Rcin450-2 primer set (internal primers, Table 1) has been devised for OsaC4H isolation. Briefly, 1 μL of KOD Plus Taq polymerase, 5 μL of 2 mM dNTP, 5 μL of 10× Taq buffer, 3 µL of 25 mM MgSO_4_, 1.5 μL each of 10 mM external primer set (Fcin450-1/Rcin450-1), 1 μL of cDNA template was added to the same tube with a final volume of 50 μL. Amplification was carried out on a programmable thermocycler (Mastercycler pro, Eppendorf, Hamburg, Germany) using the following specifications: 98 °C for 3 min, 30 cycles of 98 °C for 30 s, 42 °C for 90 s and 72 °C for 90 s, then followed by a final extension of 7 min at 72 °C. The secondary PCR consisted of 5 μL amplification product of the primary PCR using Fcin450-2/Rcin450-2 as internal primers (10 mM each). PCR buffers and reagents were identical to the primary PCR. Amplifications were performed in the same machine as described above using the following specifications: 98 °C for 3 min, and followed by 30 cycles of 30 s at 98 °C, 90 s at 42 °C and 90 s at 72 °C. This was followed by a final extension of 7 min at 72 °C. The amplicon was cloned in pEASY^TM^-T1 vector to generate pEASY-OsaC4H for sequencing. 

The pEASY-OsaC4H was used as a template for sub-cloning the full-length sequence of OsaC4H with primers FYeDP60cin450 and RYeDP60cin450 (Table 1). The PCR product was fused to the linearized pYeDP60 vector digested with *Eco*R I and *Bam*H I to yield pYeDP60-OsaC4H by In-Fusion method according to the direction contained in In-Fusion^®^ HD Cloning Kit.

### 3.7. Expression and Characterization of OsaC4H in Yeast

The pYeDP60-OsaC4H plasmid was introduced into the *S. cerevisiae* strain WHT by the LiOAc/SS carrier DNA/PEG method according to Gietz *et al.* [34]. Transformants were selected using a SD-Ade-Ura drop-out medium (SD medium without adenine and uracil). Verification of positive clones was done by extraction of yeast plasmids and further colony PCR. Yeast cultures were initially grown in 10 mL SD-Ade-Ura liquid medium at 30 °C to an OD_600_ of 2~3. 1 mL of cell was centrifuged and washed three times by ddH_2_O. The cell pellet was resuspended in 50 mL induction YPD medium containing 2% galactose and grown at 30 °C for 16~24 h. 50 mL of *S. cerevisiae* cells expressing the OsaC4H genes were harvested by centrifugation at 13,000 rpm for 5 min. The cell pellets were resuspended in 6 mL lysis buffer (50 mM Tris·HCL, pH8.0), and subjected to disrupt by high pressure (12,000 MPa, 3 times). After disruption, the debris was removed by centrifugation at 4 °C in a tabletop centrifuge (13,000 rpm, 10 min). The supernatant was used directly to carry out enzymatic functional analysis. The *in vitro* enzyme reaction was carried out in total volume of 183 µL containing 160 µL crude protein (disrupted supernatant derived 1.3 mL culture), 10 µL NADP stock buffer harboring 26 mM NADP-Na_2_, 66 mM glucose 6-phosphate and 66 mM MgCL_2_·6H_2_O, 10 µL substrate cinnamic acid (25 mM), 3 µL NADPH (125 mg·mL^−1^), 0.5 U of glucose 6-phosphate dehydrogenase. All components without substrate served as control. Reaction mixture was incubated in shaking condition (160 rpm) at 30 °C overnight and the reaction was terminated by addition of 40 μL chloroform. Denatured protein was pelleted by centrifuging for 10 min at 12,000 *g*. The product was unambiguously determined by HPLC-UV, HPLC-MS and ^1^H- and ^13^C-NMR. HPLC was performed on a HITACHI instrument using a C18 column [YMC-Pack ODS-A (5 µm, 12 nm) 250 × 4.6 mmI.D]. The reaction product was subjected to HPLC at a pump flow of 1 mL·min^−1^, column temperature at 25 °C and injection volume of 10 μL. The mobile phase was a gradient of solvent A (H_2_O containing 0.05% TFA) and solvent B (100% acetonitrile), applied as follows: 0~15 min, 10%~60% B linear; 15~16 min, 60%~100% linear; 16~23 min, 100% B isocratic; 23~24 min, 100%~10% B linear; 24~30 min, 10% B isocratic. Elution of compounds was monitored at 309 nm.

LC-MS analysis was performed on an Agilent 1200 RRLC series HPLC system (Agilent Technologies, Waldbronn, Germany) coupled to the QTRAP MS spectrometer (QTRAP 2000, Applied Biosystems/MDS SCIEX, Concord, ON, Canada) tandem mass spectrometer equipped with a Turbo Ion spray ion source which was controlled by Analyst 1.5. UV spectra were recorded from 190 to 400 nm and the detection wavelength was set at 320 nm for the enzymatic products of cinnamic acid. The mass spectrometer was operated in negative ion mode and spectra were collected in the enhanced full mass scan mode from *m/z* 100~1,000.

NMR data were obtained on a Bruker Avance III HD 600 spectrometer (Bruker, Massachusetts, USA) at 600 MHz for ^1^H-NMR and 151 MHz for ^13^C-NMR, respectively, using CDCl_3_ as solvent. Chemical shifts (δ) are given in ppm, coupling constants (*J*) are given in Hertz (Hz).

### 3.8. Phylogenetic Analysis

OsaC4H and other known C4H sequences retrieved from GenBank were aligned with CLUSTAL X 2.1. Subsequently, a phylogenetic tree was constructed using neighbour-joining (NJ) method with MEGA 5.1 software. The reliability of the tree was measured by bootstrap analysis with 1,000 replicates.

## 4. Conclusions

Herein, after sequence verification by nested RT-PCR, the products of enzymatic reactions catalyzed by recombinant OsaC4H protein were determined unambiguously by the combined use of HPLC, HPLC-MS and NMR, which showed OsaC4H was a *bona fide* C4H. OsaC4H is the first well-defined gene involved in OSW-1 biosynthesis, which has provided a first insight into the genes responsible for OSW-1 biosynthesis. Moreover, a fast method for P450 gene functional characterization was developed, including a fast, directional cloning step and functional characterization technology of P450 encoding genes without tedious microsome preparation.
